# Active Sulforhodamine 101 Uptake into Hippocampal Astrocytes

**DOI:** 10.1371/journal.pone.0049398

**Published:** 2012-11-26

**Authors:** Christian Schnell, Yohannes Hagos, Swen Hülsmann

**Affiliations:** 1 Abt. Neurophysiologie und Zelluläre Biophysik, Zentrum Physiologie und Pathophysiologie, Georg-August-Universität, Göttingen, Germany; 2 DFG Research Center Molecular Physiology of the Brain (CMPB), Göttingen, Germany; 3 Abt. Vegetative Physiologie, Zentrum Physiologie und Pathophysiologie, Georg-August-Universität, Göttingen, Germany; University of Muenster, Germany

## Abstract

Sulforhodamine 101 (SR101) is widely used as a marker of astrocytes. In this study we investigated labeling of astrocytes by SR101 in acute slices from the ventrolateral medulla and the hippocampus of transgenic mice expressing EGFP under the control of the astrocyte-specific human GFAP promoter. While SR101 efficiently and specifically labeled EGFP-expressing astrocytes in hippocampus, we found that the same staining procedure failed to label astrocytes efficiently in the ventrolateral medulla. Although carbenoxolone is able to decrease the SR101-labeling of astrocytes in the hippocampus, it is unlikely that SR101 is taken up via gap-junction hemichannels because mefloquine, a blocker for pannexin and connexin hemichannels, was unable to prevent SR101-labeling of hippocampal astrocytes. However, SR101-labeling of the hippocampal astrocytes was significantly reduced by substrates of organic anion transport polypeptides, including estron-3-sulfate and dehydroepiandrosterone sulfate, suggesting that SR101 is actively transported into hippocampal astrocytes.

## Introduction

Astrocytes are important players in neuronal networks. They maintain the extracellular milieu by removal of potassium and neurotransmitters like glutamate and modulate synaptic transmission by releasing gliotransmitters [1], [2], [3]. Unequivocal identification of astrocytes for imaging experiments as well as electrophysiological recordings was facilitated by the availability of transgenic mouse lines expressing fluorescent proteins under the control of glia specific promoters [4], [5]. Recently, sulforhodamine 101 (SR101) has been used for specific visualization of astrocytes in cortex and hippocampus [6], [7], [8], [9]. In the spinal cord, however, SR101 has been described as unspecific [10] and it was also reported to label oligodendrocytes in the rabbit retina [11]. Furthermore, during hypoxia SR101 can enter also hippocampal neurons via hemichannels [12]. Currently, it is not known how the specific loading of SR101 into astrocytes is achieved under normoxic conditions. Although the synthetic glycyrrhetinic acid derivative carbenoxolone has been shown to block SR101-labeling of astrocytes [7], it is not yet clear if this is due to its action on gap-junctions and hemichannels or a yet unknown, maybe indirect mechanism. Nevertheless, SR101 is widely used as a marker for astrocytes in different brain regions [13], [14], [15] even despite the fact that sulforhodamine 101 has been found to trigger epileptic activity in the hippocampus [16].

In this study, we initially aimed to use SR101 for identification of astrocytes in ventrolateral medulla (VLM). Since VLM astrocytes were not labeled by SR101 sufficiently, we tried to improve the staining by pharmacological manipulations of potential mechanisms that might be involved in export of SR101 from medullary astrocytes. The results guided us to a series of experiments in the hippocampus to unveil the potential mechanism and functional role of this regional heterogeneity of astrocytes in ventrolateral medulla and the hippocampus.

## Materials and Methods

### Ethics statement

In accordance with the German Protection of Animals Act (Tierschutzgesetz; TierSchG §4 Abs. 3) we did not need formal approval for the post mortem removal of brain tissue. The experiments were communicated to and notified by animal welfare office of University Medical Center Göttingen, Germany (institutional act number: T19.08).

### Breeding of mice

Animals were hold and bred in the animal facilities of the University Hospital Göttingen in accordance with guidelines of the German Physiological Society as well as the regulations of the State of Lower Saxony and the Federal Republic of Germany. Experiments were performed on acute brain slice preparations of neonatal (P2–P12), juvenile (P29–33) and adult mice (P98–99) expressing the enhanced green fluorescent protein in astrocytes [Tg(hGFAP-EGFP)GFEC-Fki; [4]]. Additionally we used mice in which glycinergic neurons were labeled [Tg(Slc6a5-EGFP)1Uze; [17]] to unequivocally identify inhibitory neurons.

### Slice preparations

Acute transversal slices from brainstem and hippocampus were prepared as described previously [18]. Briefly, animals were decapitated under diethyl-ether anesthesia, brainstem and hippocampus were isolated and placed in ice-cooled, carbogen-saturated (95% O_2_, 5% CO_2_) artificial cerebrospinal fluid (aCSF) containing 118 mM NaCl, 3 KCl, 1.5 mM CaCl_2_, 1 mM MgCl_2_, 1 mM NaH_2_PO4, 25 mM NaHCO_3_, and 30 mM D-glucose). The osmolarity was 325–335 mosm/l and the pH was adjusted to 7.4. The isolated brain part was glued with cyanoacryl glue (Loctite Deutschland GmbH) to an agar block and mounted in a vibroslicer (VT 1000S or VT 1200S, Leica). Brainstem and hippocampal slices of 250 µm were cut and stored in oxygenated aCSF at room temperature for at least 30 minutes before staining. For the actual experiments, slices were transferred to a recording chamber at the regarding microscope (see below). Slices were kept submerged by a nylon fiber grid and continuously perfused with aCSF at a flow rate of 5–10 ml/min.

### Sulforhodamine 101 staining protocol

Sulforhodamine 101 (SR101) labeling was performed with a low concentration of the dye [6], [19]. If not stated otherwise, slices were incubated for 20 minutes in carbogen-saturated aCSF containing 1 µM SR101 at 34°C. Thereafter, slices were incubated in aCSF for 10 minutes at 34°C to allow washout of excess dye from the extracellular space. Sodium-reduced solution was prepared by replacing NaCl by cholineCl.

### Fluorescence imaging using multifocal 2-photon excitation microscopy

For detection of EGFP- and SR101-fluorescence, we used multifocal (8 or 16 foci) 2-photon excitation. The principle arrangement of the microscope (TriMScope, LaVision BioTec) was described earlier [20]. Two-photon excitation was achieved with a Ti:Sapphire Laser (SpectraPhysics *MaiTai BB*) at 800 nm. Images were acquired with CCD-cameras (Ixon 885 or Clara, Andor Technology) using either no or 2×2 binning. Fluorescence signals of hGFAP-EGFP expressing astrocytes were detected through a 531/40 nm band pass emission filter, whereas SR101-fluorescence was detected through a BP 645/75 nm band pass emission filter (AHF Analysentechnik AG). Image noise minimization, was achieved either by offline averaging of four consecutive images or by integrating the emitted light of consecutive scans on the CCD camera. To allow quantitative comparison of the SR101-intensity between controls and drug treatments, all image parameters, especially camera settings (exposure time, gain and binning) as well as laser settings (laser power, beam number) were identical for a particular set of experiments. Cell counting was performed in a defined volume that was scanned with 2 µm step z-stacks using a piezo-focus (Physik Instrumente). All settings were controlled by “Imspector” software (LaVision BioTec, Bielefeld, Germany). Since fluorescence intensity of EGFP in glycinergic neurons was low and sensitive to photo bleaching, we used the dual camera mode of our microscope when performing time-lapse recordings of SR101-loading in slices from Tg(GlyT2-EGFP)-mice. In these experiments, emitted light was divided by a dichroic mirror (580 nm, beam splitter 580 DCXR, AHF), thus shorter wavelengths could be passed through a 531/40 nm band pass filter for detection of EGFP-fluorescence with a second CCD-camera (Clara, Andor Technology), while longer wavelengths (SR101-fluorescence) were detected with the other Clara CCD-camera as described above. Overlap of both cameras was adjusted before the experiment using standard fluorescent probes (Chroma). This technique allows adjusting the camera settings of the green and red channel independently and, thus, the camera setting for detection of SR101 could remain the same as in the single camera experiments.

For analysis of SR101-fluorescence, “Imspector”-images were exported to TIFF-format. Deconvolution was performed with *Autoquant* software (MediaCybernetics) using the theoretical point-spread-function (adaptive PSF, 10 iterations). 3D-volumes were then further analyzed in *Imaris* (Bitplane). Drift correction was performed for time-lapse recordings. The fluorescence intensity of the SR101 and EGFP channel was determined using the spot objects feature of *Imaris*. A spherical 3D volume (spot) of 6 µm diameter was manually assigned to EGFP-expressing cells in the “surpass” view. In the SR101-channel the “recenter” function of *Imaris* was used to identify cells with SR101-labeling. Only if “recentering” was possible a cell was counted as a SR101-positive. To quantify the fluorescence intensity of an individual cell the median intensity of the assigned spot was calculated (in the time-lapse experiments for each time point). We used a *R* script (The R Foundation for Statistical Computing) to extract the number of cells and their intensities out of the comma separated file that was exported from *Imaris* and to calculate the average SR101-fluorescence intensities of the regarding slice.

### Electrophysiology

Electrophysiological characterization of SR101-labeled or EGFP-labeled CA1 *stratum radiatum* cells was performed as described earlier [9], [21]. After SR101-staining, using the protocol described above, hippocampal slices were transferred to a custom-built recording chamber mounted to an upright microscope (Axio Examiner.Z1, Zeiss) and superfused with aCSF at room temperature. Epifluorescence illumination was achieved by a HBO100 mercury lamp (Zeiss). Two filter sets were used to discriminate between SR101-labeled cells that did not express EGFP and EGFP-expressing astrocytes. A dualband GFP/mCherry ET filter set (F56-019; AHF Analysentechnik) allowed the identification SR101-positive cells while the GFP filter set (38; AHF) was used to confirm the expression of EGFP. Fluorescence illumination was stopped to avoid bleaching, and a Dodt-Gradient-Contrast [22] was used to approach the identified cell with the patch-pipette. EGFP- and SR101-fluorescence was documented using a CCD-camera (Sensicam QE; PCO) that was controlled by CamWare software (PCO).

Whole-cell recordings were conducted with a Multiclamp 700A amplifier, Digidata 1440A interface and pClamp10 software (Molecular Devices, Forster City, CA, USA). For characterization of the current-voltage (I–V) relations, SR101-positive and EGFP-positive cells that were voltage-clamped at a holding potential of −80 mV, were exposed to 200 ms voltage-steps that reached from −160 mV to +60 mV (10 mV increments). Current responses were low-pass filtered at 2 kHz and digitized at 10 kHz. The steady state current at 200 ms was measured to calculate the (I–V) curves. Additionally, the membrane resistance was calculated from the change of the holding current in response to a hyperpolarizing voltage step to −90 mV.

### Drugs

Electrolytes for aCSF (see above) were purchased from Sigma-Aldrich (Taufkirchen, Germany) and Merck chemicals (Darmstadt, Germany). Drugs were stored in concentrated stock solution at −20°C and used following dilution in aCSF in final concentrations as follows: Carbenoxolone (CBX, 100 µM, Sigma-Aldrich), Probenecid (1 mM, Sigma-Aldrich), Estrone-3-sulfate (E3S, 100 µM, Sigma-Aldrich), MK-571 (50 and 200 µM, Enzo Life Sciences), Rifampicin (100 µM, Sigma-Aldrich), Dehydroepiandrosterone sulfate (DHEAS; 100 µM, Sigma-Aldrich). Mefloquine (MFQ) was purchased from BioBlocks as (±)-erythro-(R*/S*)-mefloquine (QU024-1) and used in concentrations between 0.1–50 µM.

### Data analysis

Data are expressed as mean ± SEM. Statistical comparison was performed with the SigmaPlot software (Systat Software, Inc.). Statistical significance (t-test or Mann-Whitney U test) was expected if p<0.05.

## Results

### Sulforhodamine 101 is not a selective marker for astrocytes in the ventrolateral medulla

**Figure 1:**
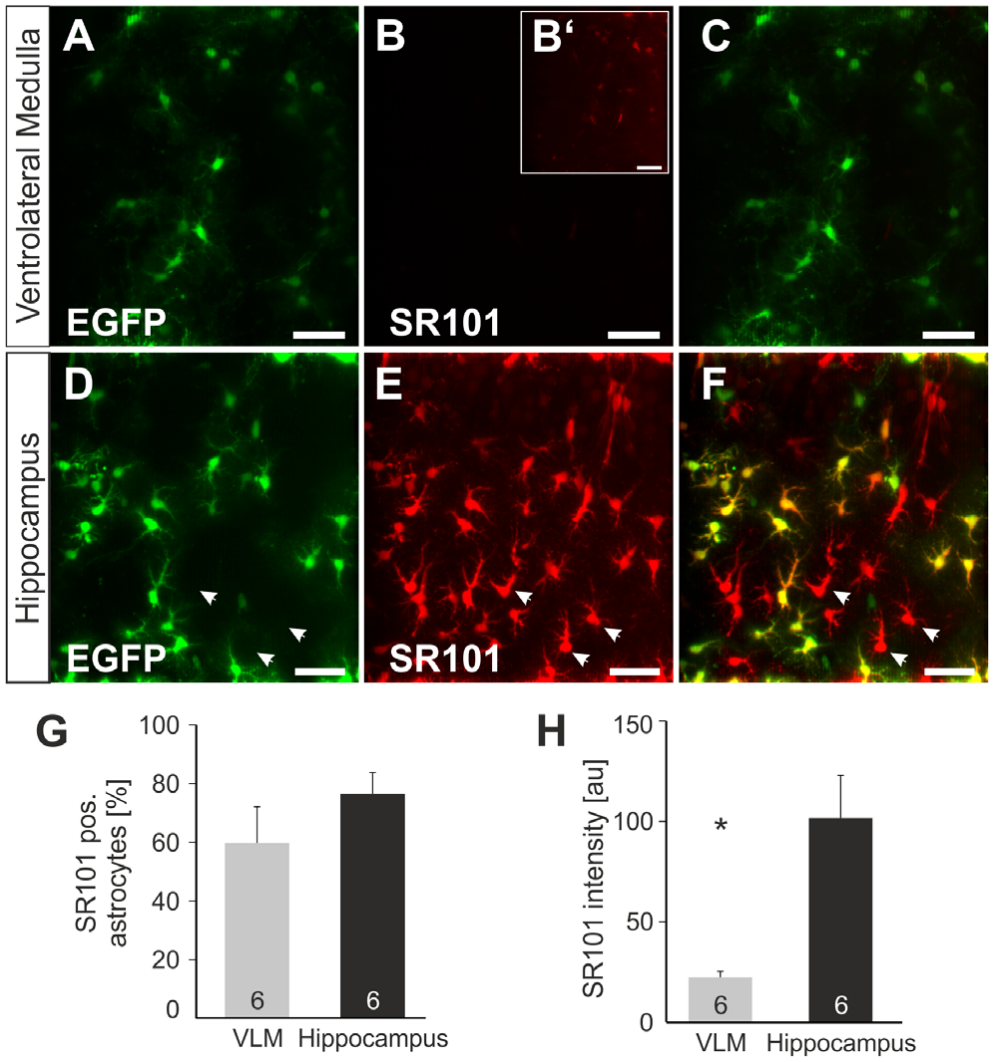
Sulforhodamine 101 labeling in the ventrolateral medulla (VLM) and *stratum radiatum* (CA1 area) of the hippocampus. **A–C:** Sulforhodamine (SR101) labeling (**B**) in VLM from transgenic mice expressing EGFP (**A**) under the hGFAP-promotor. **B′**: The overall labeling with SR101 of the VLM is very weak and only visible after increasing image gain. **C**: Overlay of A and B. **D–E:** In hippocampal slices, SR101-staining (**E**) largely overlaps with EGFP-labeling (**D**) of astrocytes. (**F**) Overlay of D and E. Images are maximum intensity projections of 21 2P-images, 2 µm distance). Scale bars: 40 µm. **G–H:** Statistical comparison of labeling of EGFP-astrocytes with SR101 in slices from the VLM and hippocampus. **G:** Comparison of the percentage of EGFP-positive astrocytes that showed detectable levels of red SR101-fluorescence. **H:** Comparison of SR101-intensity of EGFP-positive astrocytes. The intensity of SR101 is significantly reduced (asterisk) in the VLM. Error bars = standard error of the mean.

To assess the specificity of SR101-staining in the ventrolateral medulla (VLM) in the brainstem, we adopted the Sulforhodamine 101 (SR101) staining protocol described earlier for acute hippocampal slices [6], to acute brainstem slices from neonatal Tg(hGFAP-EGFP) mice (Figure 1). It was obvious that labeling of VLM-astrocytes with SR101 was not sufficient for the reliable identification of astrocytes (Figure 1B). The intensity of SR101-staining was so weak that we had to increase the gain of the images to detect SR101-staining in astrocytes at all (Figure 1B′). In contrast, SR101-staining of hippocampal slices with the same protocol confirmed a good labeling of *stratum radiatum* astrocytes in the CA1 and CA3 region with SR101 (Figure 1F).

We quantified the differences in SR101-labeling by measuring the intensity of the red-fluorescence using 2-photon microscopy. In the VLM we were able to identify a faint SR101-fluorescence, which was different from the background, in 59.9±12.4% EGFP-positive astrocytes. The SR101-intensity was determined to be 22.32±2.9 au (n = 5 slices; mean ± SEM). In the hippocampus SR101-intensity of astrocytes (EGFP-positive) was five-fold higher (101.72±21.29 au) (n = 6, p<0.05, Mann-Whitney U test). The number of EGFP-positive cells that were stained with SR101 was 76.5±7.2% (n = 6, n.s., t-test).

**Figure 2:**
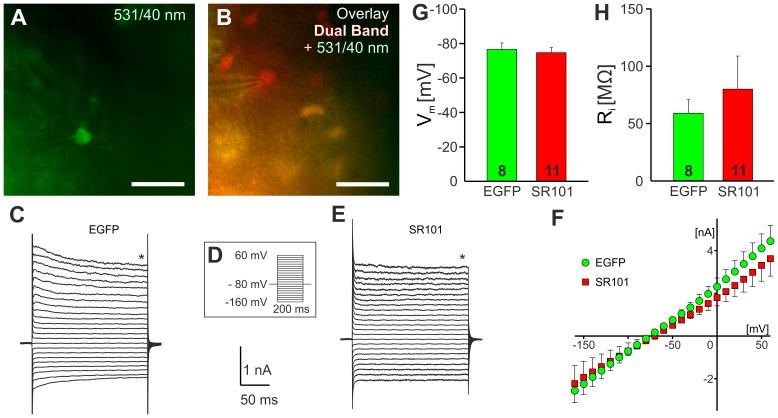
Electrophysiological characterization of SR101-positive cells in the hippocampus. **A:** CCD-camera image of an EGFP-expressing astrocyte using a EGFP-filter, 531/40 nm BP filter. The image was taken after the cell was approached with patch-pipette. **B:** Image of a whole-cell recorded SR101-loaded *stratum radiatum* cell that did not express EGFP. The picture was merged from the EGFP-filter image (531/40 nm, green) and the dual band filter image (EGFP/mCherry; F56-019, red). **C:** Membrane current traces of the astrocytes in (A) in response to the voltage-step protocol shown in **D**. **E:** membrane current traces of the SR101-positive EGFP-negative cell in (B). Capacitance artifacts have been truncated. **F:** Averaged I–V curves from EGFP-positive (EGFP; green) and SR101-positive EGFP-negative (SR101; red) cells. **G,H:** Statistical comparison of resting membrane potential (G) and membrane resistance (H).

In the hippocampus 46.3±3.1% of SR101-positive cells were lacking EGFP-fluorescence. These cells resembled astrocytes in size and shape and when we recorded from these cells we found no differences in the electrophysiological properties between EGFP-negative SR101-positive cells and EGFP-positive astrocytes (Figure 2). The resting membrane potential of EGFP-positive astrocytes was −76.63±3.74 mV (n = 8) and −74.73±2.93 mV (n = 11) for the SR101-positive but EGFP-negative cells. No difference was detected in the membrane resistance (SR101-positive but EGFP-negative: 80.1±28.8 MΩ; EGFP-positive: 59.0±11.9 MΩ). Additionally, it appears that SR101-labeled cells that did not express EGFP resembled also mature astrocytes indicated by the almost linear IV-curve (Figure 2H).

**Figure 3:**
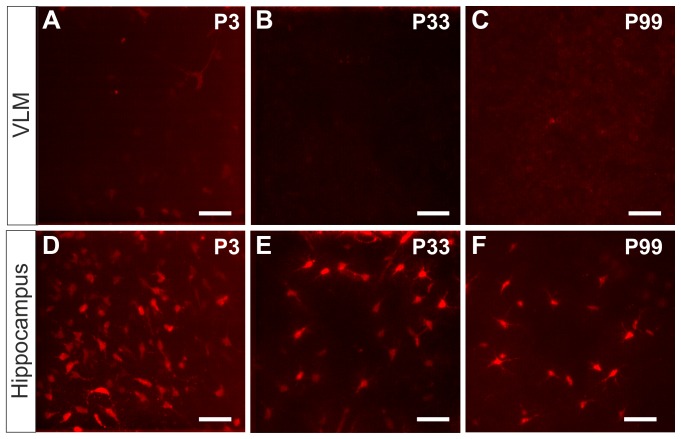
Comparison of SR101-staining in different ages. **A–C:** staining in the ventrolateral medulla (VLM) with 1 µM SR101 for 20 min and 10 min wash out. EGFP-fluorescence is not shown. The SR101 labeling was poor in all three tested ages. **D–F:** In the *stratum radiatum* of the hippocampal CA1 region SR101-labeling of juvenile (P33) an adult mice (P99) was very similar as compared to neonatal mice.

We also performed SR101-labeling in brainstem and hippocampus slices from different developmental stages (Figure 3). While in the VLM SR101 did not allow a reliable identification of astrocytes at any time (Figure 3A–C), solid SR101-staining of astrocytes was always possible in the *stratum radiatum* of the CA1 region (Figure 3D–F).

### 2-photon time lapse imaging reveals transient labeling of non-astrocytic cells in the VLM

**Figure 4:**
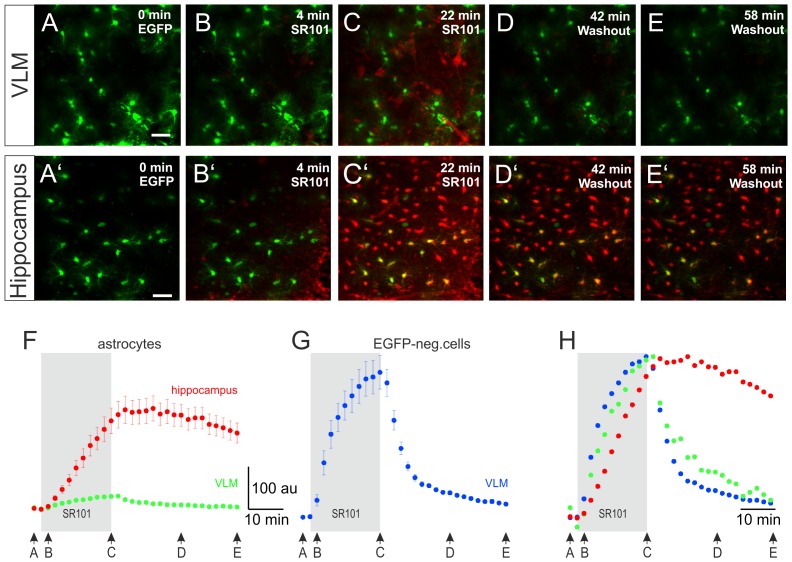
Time course of the SR101-staining procedure. (**A–E′**) Images show 2-photon time-lapse recording of SR101-stainings and unstaining during washout in acute slices (40 µm, 21 images, 0.5 min^−1^). **A–E** show the results from the ventrolateral medulla (VLM) and **A′–E′** from hippocampus. **A, A′:** Maximum intensity projections of the EGFP-fluorescence of the astrocytes. **B–E, B′–E′:** Images show maximum intensity projections of the SR101-labeling at 4 different time points in the hippocampus and in the VLM. Scale bars: 40 µm. **F–H:** Analysis of the time course of SR101-staining. Arrows underneath the traces represent the time points according to the images in A–E and A′–E′, respectively. Data (mean ± SEM) is derived from 3–5 cells per slice (6 slices in hippocampus and 5 in the VLM). **F:** Note that the SR101-staining of hippocampal astrocytes (red) is much stronger than the staining in the VLM astrocytes (green). **G:** In the VLM additionally EGFP-negative cells are stained while SR101 is applied (blue trace) but the fluorescence is disappearing during the washout. **H:** Normalized time course of the staining of astrocytes (red in the hippocampus; green in the VLM) and EGFP-negative cells in the VLM (blue).

To examine the time course of SR101-labeling of astrocytes, we performed time-lapse 2-photon-imaging during the labeling procedure. After recording the fluorescence background in aCSF for 2 min, SR101 (1 µM) was bath-applied. Then changes of the fluorescence intensity were recorded every 2 min. In the VLM, EGFP-positive astrocytes did not take up much SR101 during the 20 min loading phase (Figure 4 C). However, we found SR101-positive cells that were EGFP-negative (Figure 4 C,G). During washout, SR101-fluorescence disappeared rapidly from the latter (Figure 4 F,G).

In the hippocampus most EGFP-positive astrocytes but also EGFP-negative were loaded with SR101 during the 20 min loading phase (Figure 4 C′). Among the EGFP-negative cells most SR101-labeled cell resembled the EGFP-positive astrocytes in size and shape. During washout, SR101-fluorescence of EGFP-positive astrocytes decreased only slightly and allowed identification of astrocytes until the end of the recording (Figure 2 D′,E′). Additionally, a large number of the EGFP-negative cells retained SR101, yet, most of them resembling astrocytes in size and shape, confirming the labeling pattern of the initial staining experiments described above.

**Figure 5:**
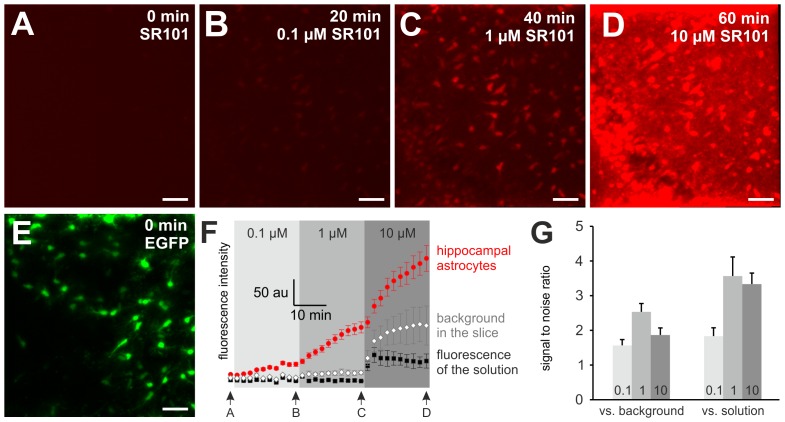
Accumulation of SR101 in cells. **A–D:** Maximum intensity projections of 2-photon z-stacks from time-lapse recordings during the staining of hippocampal CA1 region with different SR101-concentrations (70 µm z-stacks, 2 µm steps, 2 min^−1^). **A:** SR101-fluorescence before SR101 was applied. **B–D:** SR101-fluorescence at different time points as indicated. **E:** EGFP-fluorescence of the astrocytes. Scale bars: 40 µm. **F:** Quantitative analysis of the time course of the SR101- fluorescence in hippocampal astrocytes (n = 17 astrocytes, 3 slices, red circles) and SR101-intensity of the background within the slice (n = 3 spots) and in the SR101 containing aCSF solution above the slice (n = 3 spots). **G:** Comparison of signal to noise ratio of the somatic SR101-fluorescence of astrocytes versus background (in the slice) and versus the SR101 in the aCSF solution above the slice. Note: 1 µM SR101 gave the best signal to noise ration.

In an additional set of 2-photon time lapse imaging experiments, we stepwise increased the SR101-concentration in the bath solution (Figure 5; n = 3). With a SR101 concentration of 0.1 µM, astrocytes in the CA1 *stratum radiatum* started to become labeled (Figure 5 B). During the 20 min staining procedure the SR101-fluorescence intensity in the astrocytes was increasing above the fluorescence intensity of the SR101-containing aCSF that was measured above the slice (Figure 5F). At 1 µM, the intracellular SR101-intensity increased further (Figure 5 C,F), while also the SR101 background fluorescence in the slice increased slightly. Both measured fluorescence intensities were larger as compared to the SR101-fluorescence in solution above the slice. Subsequent increase of the SR101-concentration to 10 µM caused an additional increase of the SR101-fluorescence of astrocytes during the 20 min staining procedure. The fluorescence intensity at 20 min did not reach a plateau, suggesting continuing accumulation of SR101 in the astrocytes (Figure 5D). In all 3 tested conditions the SR101-intensity in astrocytes exceeded the fluorescence intensity of the background in the slice as well as the intensity in the aCSF (See also Movie S1). However the best signal to noise ratio was found in the 1 µM SR101-solution (Figure 5G).

### Gap-junction hemichannels are not responsible for SR101-uptake in the hippocampus

**Figure 6:**
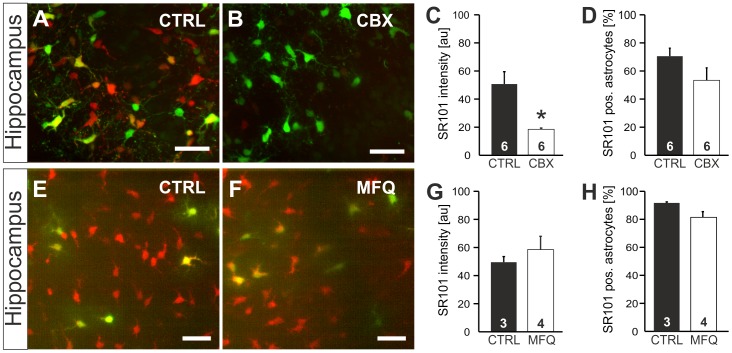
Effects of the hemichannel blocker carbenoxolone and mefloquine on SR101-labeling in the hippocampus. **A–D:** Carbenoxolone (CBX) reduced SR101-labeling in the hippocampus. **A:** Overlay of EGFP (green)- and SR101-fluorescence (red) in CTRL conditions. **B:** With CBX (100 µM) present during SR101-staining, SR101-fluorescence is significantly reduced. **C,D:** Statistical comparison of SR101-labeling of EGFP-labeled astrocytes in the absence (CTRL) and presence of CBX reveals significant reduction of fluorescence intensity but no significant difference in the fraction of SR101-positive astrocytes. **E:** Overlay of EGFP- and SR101-fluorescence after a staining in CTRL conditions. **F:** Overlay of EGFP- and SR101-fluorescence after a staining with Mefloquine (MFQ, 50 µM). **G,H:** Statistical comparison: Neither the staining intensity (G) nor the number EGFP-positive astrocytes that were labeled with SR101 (H) was changed. Scale bars: 40 µm.

In the original study describing the specific labeling of astrocytes with SR101 *in vivo* [7], the gap-junction blocker carbenoxolone was found to suppress labeling of astrocytes by topical applied SR101. Thus one can speculate that SR101 enters hippocampal astrocytes via gap-junctional hemichannels. We therefore tested if carbenoxolone also blocks SR101-labeling of hippocampal astrocytes in the slice preparation. Carbenoxolone (CBX; 100 µM) reduced SR101-labeling of EGFP-positive astrocytes (50.61±21.59 au (CTRL) vs. 18.35±2.16 au (CBX); n = 6, p<0.05, Mann-Whitney U test; Figure 3A–C). The fraction of SR101-positive EGFP-positive astrocytes was, however, not reduced by CBX (70.5±5.7% (CTRL) vs. 53.4±8.9% (CBX); n = 6, n.s., t-test; Figure 6D).

Carbenoxolone does not discriminate between hemichannels formed by connexins or pannexins [23]. Therefore we tested if the anti-malaria drug and pannexin blocker mefloquine (MFQ) blocks SR101-staining of hippocampal astrocytes. However, SR101-staining of hippocampal astrocytes was not changed by MFQ using a concentration of 0.1 µM or 1 µM (not shown). Even at a concentration of 50 µM when MFQ blocks also connexin hemichannels [24] no change of SR101-intensity (58.5±9.5 au in MFQ (n = 4) vs. 49.5±4.0 au in CTRL (n = 3, n.s., t-test, Figure 6G) or cell number (81.7±4.1% in MFQ (n = 4) vs. 92.0±0.7% in CTRL (n = 3, n.s., t-test, Figure 6H) was observed.

Since MFQ did not affect SR101-fluorescence intensity, we conclude that hemichannels formed by connexins or pannexins are not the major route for SR101-uptake and that the organic anion carbenoxolone might interfere with another uptake mechanism.

### MK-571 reduces SR101-labeling in the hippocampus

Time-lapse imaging suggested that SR101 might be removed from neurons and VLM astrocytes via an active transport mechanism. Possible candidates are multidrug resistance proteins, which can mediate efflux of SR101 from cells [25] and also have been found in astrocytes [26]. Furthermore, blockade of efflux-transporters was shown to improve fluorescent dye-labeling of neurons [27].

**Figure 7:**
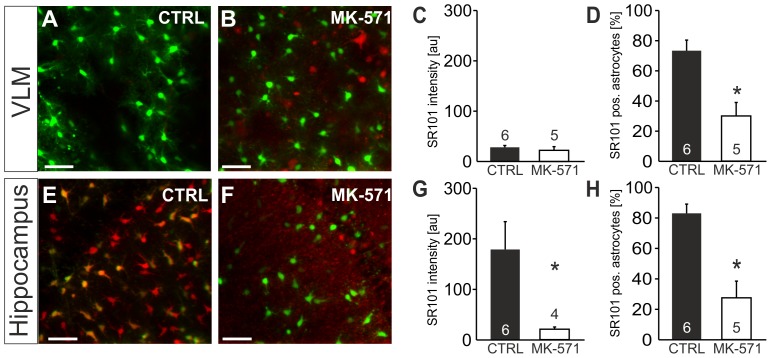
Test for the contribution of MRP-transporters to selective SR101-labeling. **A–H:** Effect of Mrp1-blocker MK-571 on SR101-labeling in the VLM (**A–D**) and hippocampus (**E–H**) using EGFP-expressing astrocytes. **A:** Overlay of EGFP- and SR101-fluorescence in the VLM after staining in CTRL conditions. **B:** Overlay of EGFP- and SR101-fluorescence after staining with MK-571 (200 µM) in the staining solution. **C, D:** Statistical comparison shows no change of SR101-fluorescence intensity of EGFP-positive astrocytes in the VLM (**C**) but a reduction of the fraction of EGFP-positive astrocytes that were also labeled with SR101. **E:** Overlay of EGFP- and SR101-fluorescence in the hippocampus after staining in CTRL conditions. **F:** Overlay of EGFP- and SR101-fluorescence after staining with MK-571 (200 µM) in the staining solution. **G, H:** Statistical comparison reveals that the intensity of SR101-labeling as well as the fraction of EGFP-positive astrocytes that were also labeled with SR101 was significantly reduced by MK-571 present during staining.

When we tested MK-571 (200 µM), an inhibitor of the Mrp1 (ABCC1) transporter, SR101-labeling of EGFP-positive VLM-astrocytes was not improved (Figure 7, A–D). The SR101 intensity was 22.2±7.4 au (n = 5) in MK-571 (vs. 28.3±3.5 au in aCSF; n = 6, n.s., t-test, Figure 7C). Rather, the number of EGFP-positive astrocytes, in which some SR101-fluorescence could be detected was reduced (30.1±8.9% (n = 5) in MK-571 vs. 73.4±7.0% in aCSF; n = 6, p<0.05, t-test, Figure 7D). Interestingly, labeling of EGFP-negative cells, including some that rather looked like neurons (arrow in Figure 7B), appeared to be improved by MK-571 in the VLM.

When we applied MK-571 to hippocampal slices, the number of SR101-positive astrocytes was significantly reduced (27.6±11.0% (n = 5) in MK-571 vs. 83.1±6.0% in aCSF; n = 6, p<0.05, Mann-Whitney U test, Figure 7H). The effect of MK-571 on the SR101-intensity of EGFP-positive astrocytes was even more pronounced (21.3±4.3 au (n = 4) in MK-571 vs. 179.1±55.0 au in aCSF; n = 6, p<0.05, Mann-Whitney U test, Figure 7G). One possible interpretation of this observation is that MK-571 interferes with the uptake of SR101.

### Neurons can be labeled by SR101 in the ventrolateral medulla

**Figure 8:**
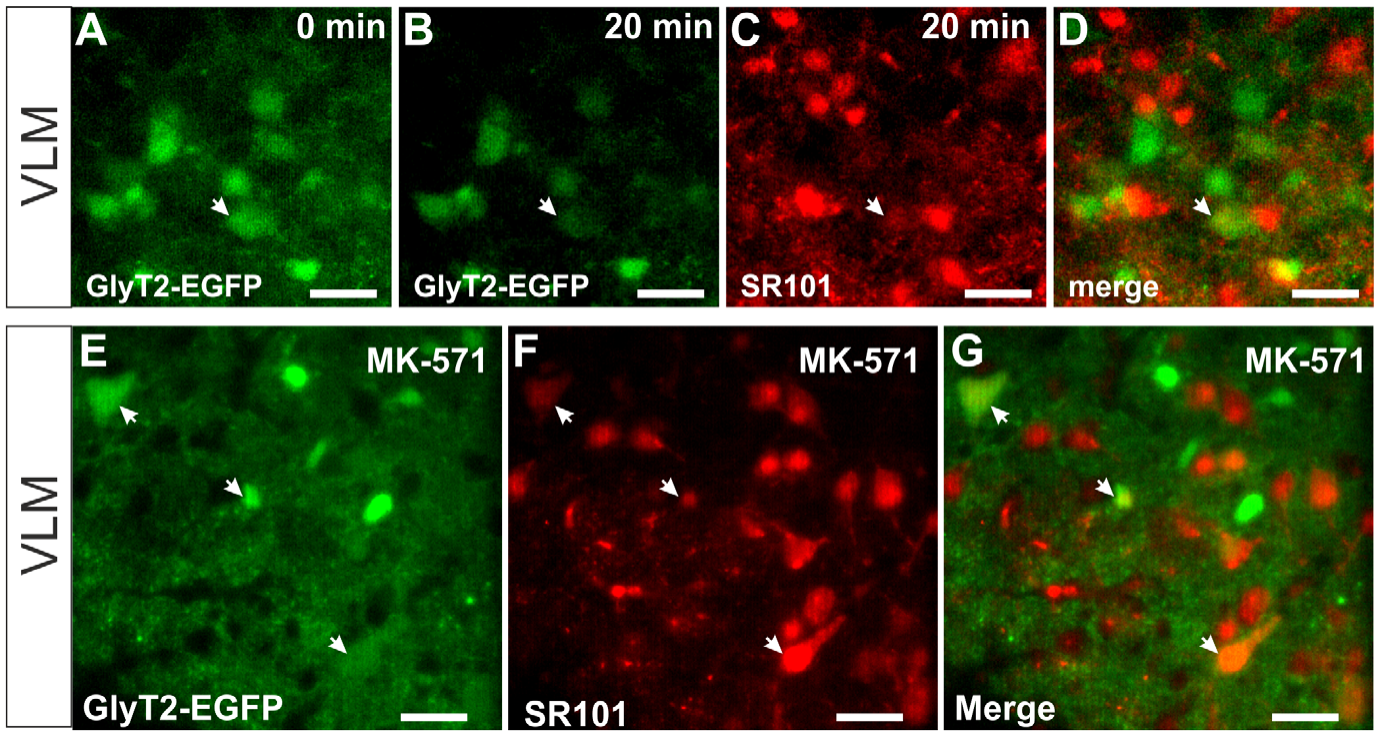
SR101 in neurons of the ventrolateral medulla. **A–D:** two-photon images from the SR101-labeling procedure of EGFP-labeled inhibitory neurons (GlyT2-EGFP) in the ventral-lateral medulla. **A,B:** Green EGFP-fluorescence is quickly photo-bleached during the 20 min SR101 application. **C:** At 20 min, SR101 is increasing in numerous cells, but SR101 could only be found in one EGFP-positive neuron (arrow). **D:** Overlay of panels A and C. **E–G:** SR101-labeling of GlyT2-EFGP neurons (**E**) and SR101-labeled cells (**F**) in the presence of MK-571 (200 µM). **G:** merged image from E and F. The arrows point to SR101-labeled glycinergic neurons. Scale bars: 40 µm.

In the VLM, as shown in Figure 4, SR101 enters EGFP-negative cell when SR101 is applied but leaves these cells once SR101 is removed from the extracellular medium. When MK-571 is applied in the VLM, SR101 was also detectable in EGFP-negative cells (Figure 7), which suggests that extrusion might be depending on a MK-571-sensitive process. Since some of the EGFP-negative SR101-positive cells had the shape and size of neurons, we aimed to test this by using another transgenic mouse line that expresses EGFP under the control of the GlyT2-promotor [28]. In a first set of experiments we performed time lapse imaging of the labeling process with 1 µM SR101 in the VLM (Figure 8A–D). Most of the SR101-fluorescence did not co-localize with cell bodies of glycinergic neurons. We could measure SR101-fluorescence after 20 min of SR101-application in only 2 glycinergic neurons (n = 3 slices). When we labeled VLM-slices (n = 5) from GlyT2-EGFP mice in the presence of MK-5171 (200 µM) we also found SR101 in a small number of glycinergic neurons (Figure 8 E–G). On average 7.3±2.7% of the SR101-positive cells were glycinergic neurons.

### Probenecid reduces astroglial SR101-uptake into hippocampal astrocytes

**Figure 9:**
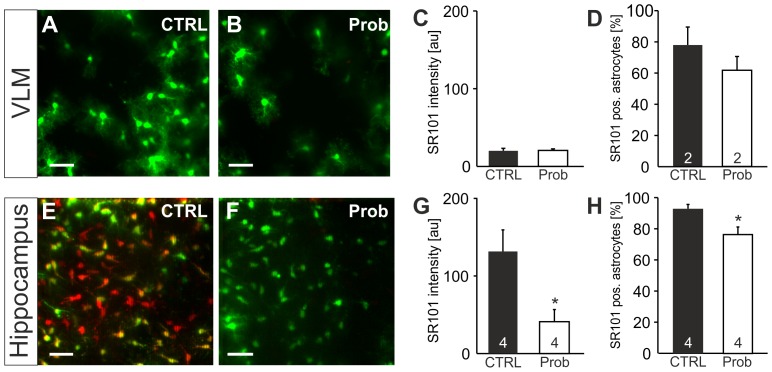
Effect of probenecid on SR101-labeling. ***A:*** Overlay of astroglial EGFP-fluorescence and SR101-fluorescence in the VLM after staining in CTRL conditions. **B:** Overlay of astroglial EGFP- and SR101-fluorescence after staining with Probenecid (Prob, 1 mM). **C,D:** Statistical comparison shows no change of astroglial SR101-labeling in the VLM. **E:** Overlay of astroglial EGFP- and SR101-fluorescence in the hippocampus after staining in CTRL conditions. **F:** Overlay of astroglial EGFP- and SR101-fluorescence after staining with Probenecid present in the staining solution shows a reduction of astroglial SR101-labeling in hippocampal astrocytes. **G,H:** Statistical comparison reveals a significant reduction of the intensity (G) as well as the percentage of EGFP-positive astrocytes that were also labeled (H). Scale bars: 40 µm.

Probenecid [29], another MRP-transporter blocker, also did not increase the SR101 labeling in VLM astrocytes (Figure 9A–D). This indicates that the weak SR101-labeling of astrocytes in the ventrolateral medulla is not due to an active extrusion mechanism but rather result of a poor uptake. Like MK-571, probenecid reduced the SR101-labeling of hippocampal astrocytes. The SR101-intensity of EGFP-positive astrocytes was reduced by probenecid (1 mM) to 41.3±15.6 au (n = 4) as compared to 131.8±27.8 au in aCSF (n = 4, p<0.05, t-test, Figure 9G). In probenecid, the fraction of SR101-positive astrocytes was smaller (76.5±4.9% (n = 4)) as compared to aCSF (93.3±2.3%; n = 4, p<0.05, t-test, Figure 9H). Since it is very unlikely that MRP-transporter (e.g. ABCC1 transporter) mediate an uptake into cells [30], our observation might be explained by an interaction of probenecid and MK-571 with another type of uptake transporters.

### SR101 labeling can be reduced by antagonists of organic anion transporting polypeptides

**Figure 10:**
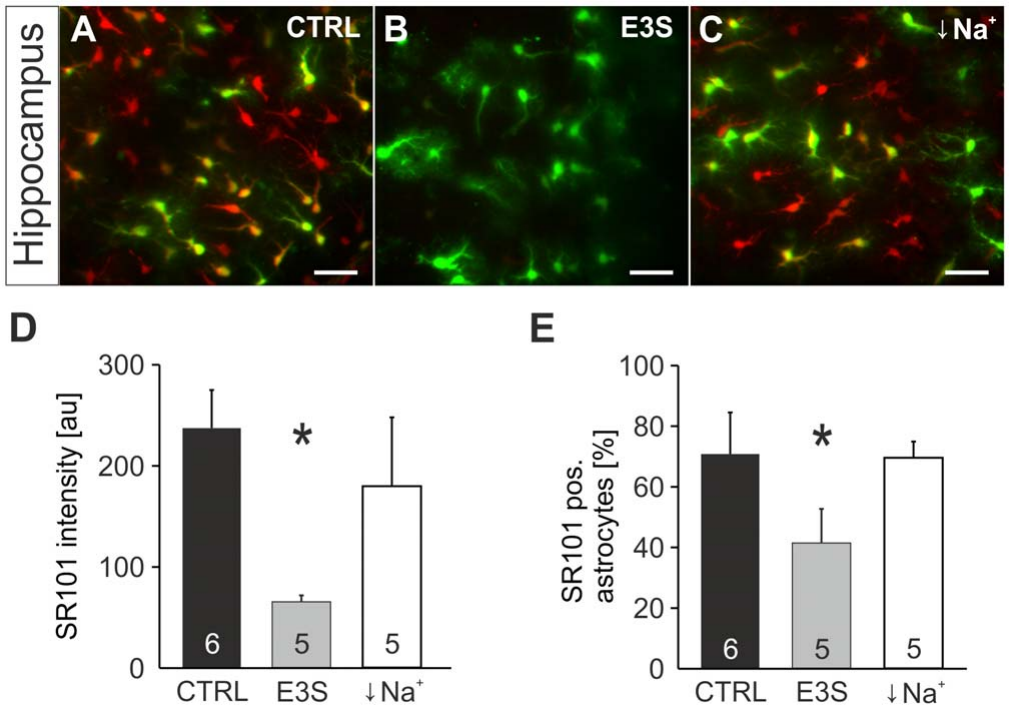
Effects of Estrone-3-Sulfate and Na^+^-free solution on SR101-uptake by astrocytes. **A:** Overlay of astroglial EGFP- fluorescence (green) and SR101-fluorescence (red) in the hippocampus after a staining in CTRL conditions. **B:** Overlay of EGFP- and SR101-fluorescence after application Estrone-3-Sulfate (E3S, 100 µM) in the staining solution shows significant reduction of SR101-fluorescence intensity. **C:** Overlay of EGFP- and SR101-fluorescence in the hippocampus after staining in Na^+^-free solution shows no Na^+^-dependence of SR101-uptake. Scale bars: 40 µm. **D:** the statistical comparison confirms significant reduction of SR101-labeling by E3S in the hippocampus, but no effect of Na^+^-free solution. (**E**) Statistical comparison of the effects of E3S and Na^+^-free solution on the percentage of SR101-labeled astrocytes in the hippocampus.

Probenecid is not only known to block MRP-transporters but is also a blocker of organic anion transporters (OATs) [31], [32] and some organic anion transporting polypeptides (OATPs) [33]. Since SR101 is an organic anion [34], [35], we hypothesized that OATs and OATPs are candidates for the SR101-uptake into hippocampal astrocytes. By simultaneous application of estrone-3-sulfate (E3S; 100 µM), a substrate of both families of organic anion transporters, we observed a strong reduction of SR101-intensity in hippocampus astrocytes (65.5±6.4 au (n = 5) as compared to 237.3±37.7 au in aCSF (n = 6, p<0.05, Mann-Whitney U test, Figure 10D). The fraction of SR101-positive astrocytes was also reduced (68.1±9.4% (n = 5) in E3S vs. and 87.3±2.4% in aCSF (n = 6, p<0.05, Mann-Whitney U test, Figure 10E). Since E3S is also a substrate of sodium-dependent anion transporters of the SLC10 family [36], we tested SR101-uptake in sodium free aCSF. However, no significant reduction of SR101-staining was observed (Figure 10C–E).

**Figure 11:**
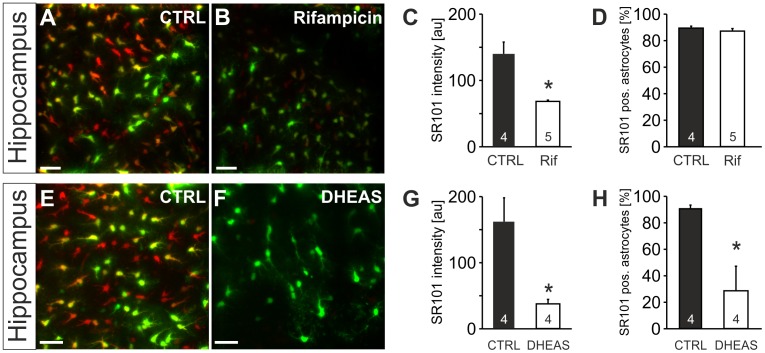
Effects of OATP blockade on SR101-uptake in the hippocampus. **A:** Overlay of astroglial EGFP-fluorescence (green) and SR101-fluorescence (red) in the hippocampus after a staining in control conditions. **B:** Rifampicin in the staining solution decreased SR101-fluorescence intensity. **C:** While the reduction of SR101-labeling by rifampicin (Rif; 100 µM) was significant, the fraction (**D**) of SR101-labeled astrocytes remained unchanged by rifampicin. **E:** astroglial EGFP-fluorescence (green) and SR101-fluorescence (red) in the hippocampus after a staining in control conditions. **F:** Reduced SR101-labeling of hippocampal astrocytes when the neurosteroid dehydroepiandrosterone sulfate (DHEAS, 100 µM) was present during SR101-staining. **G:** The statistical comparison reveals a significant reduction of SR101-fluorescence intensity by DHEAS **G:** Additionally, the percentage of EGFP-positive astrocytes that were also labeled with SR101 was significantly lowered as compared to control. Scale bars: 40 µm.

Furthermore, rifampicin (100 µM; Figure 11), which is an inhibitor of OATPs but not of OATs [32] reduced the SR101-intensity of hippocampal astrocytes to approximately 50% (Figure 11C), while leaving the fraction of labeled astrocytes unchanged.

### The OATP substrate DHEAS reduces SR101-uptake

The brain endogenous steroid hormone dehydroepiandrosterone sulfate (DHEAS; [37]) has been shown to be a substrate of OATPs [38]. When we tested the effect of 100 µM DHEAS on SR101-uptake, we observed a significant reduction of the SR101-fluorescence of hippocampal astrocytes (37.7±6.8 au in DHEAS (n = 4) vs. 162.1±36.1 au in CTRL (n = 4), p<0.05, Mann-Whitney U test, Figure 11F,G). We observed also a significant reduction of the fraction of SR101-labeled EGFP-positive astrocytes (28.7±18.5% in DHEAS (n = 4) vs. 90.6±2.8% in CTRL (n = 4), p<0.05, t-test, Figure 11H). This strong reduction of astroglial SR101-labeling indicates that DHEAS competes with the uptake of SR101 into astrocytes.

## Discussion

SR101 is an excellent tool for the identification of astrocytes for imaging experiments in the hippocampus and cortex of rodents [6], [7]. In this study, we show that SR101 is taken up into hippocampal astrocytes via an active transport mechanism. The uptake is likely to be mediated via a yet not described (orphan) organic anion transporting polypeptide (OATP; SLCO family), which can be blocked by probenecid and rifampicin.

### SR101 is not a good marker for astrocytes in the ventrolateral medulla

We were unable to stain astrocytes efficiently in the ventrolateral medulla with SR101. SR101-labeling of astrocytes was also poor in the hypoglossal nucleus and in the spinal trigeminal nucleus (data not shown). In the ventrolateral medulla, SR101 can enter neurons (Figure 8) and other cell types during the staining procedure but escapes from these cells when the slice is rinsed with normal aCSF (Figure 2). However, additional experiments are necessary to clarify the mechanism of SR101-loading in neurons. In this context it is noteworthy that, during hypoxia, hippocampal neurons can be labeled with SR101 via gap-junction hemichannels [12].

### SR101 labels astrocytes in the hippocampal stratum radiatum efficiently and selectively

We found that about 46% of the SR101-positive cells did not express EGFP. However, SR101-positive EGFP-negative cells in the hippocampus resemble astrocytes in shape, size and process morphology, corroborating earlier observations by other groups [6], [8]. When we performed whole-cell recordings from SR101-positive EGFP-negative cells in the *stratum radiatum* of the hippocampus we only found cells that had electrophysiological properties of astrocytes (Figure 2). Thus we conclude that SR101 is an efficient and selective marker for hippocampal astrocytes.

### Blockers of organic anion uptake prevent SR101-labeling of hippocampal astrocytes

In the hippocampus astrocytes, intracellular SR101-concentration reaches a much higher level compared to the VLM (Figure 4). Obviously, the SR101-fluorescence within the astrocytes increased above the fluorescence intensity of the SR101 containing aCSF, suggesting that SR101-fluorescence intensity is not depending on passive diffusion but rather on an active SR101-uptake into hippocampal astrocytes.

Since carbenoxolone [39] and probenecid are organic anions and probenecid is known to block organic anion transport [40], possible transporters for the SR101-uptake are organic anion transport systems including organic anion transporters (OAT) belonging to the SLC22 family or organic anion transporting polypeptides (OATP; SLCO family) and also transporters of the SLC10 family. Estron-3-sulfate (E3S) is the substrate of many members of these transporter families [32] and indeed was able to reduce the SR101-labeling significantly. Interestingly, organic anion transporting polypeptides have also been shown to be inhibited by MK-571 [41] and probenecid [40]. Finally rifampicin (rifampin) is known as an inhibitor of OATPs, i.e. OATP1A2, OATP1B1, OATP1B3, and OATP2B1 but not for OATs [32], strongly suggesting that SR101-uptake is mediated via an organic anion transporting polypeptide.

**Figure 12:**
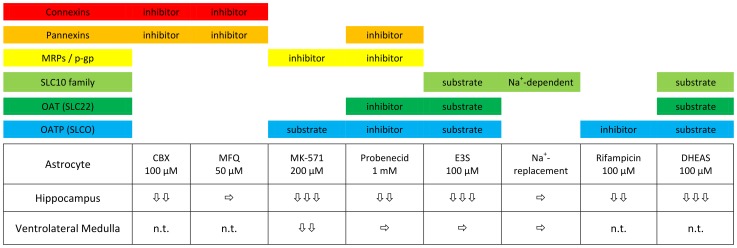
Pharmacological profile of astrocyte SR101-staining. Carbenoxolone (CBX); mefloquine (MFQ); estrone-3-sulfate (E3S); dehydroepiandrosterone sulfate (DHEAS); not tested (n.t.); very strong reduction of intensity (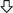 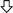 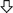), strong reduction (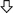 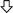), moderate reduction (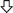), unchanged (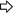).

Although little is known about OATP expression in astrocytes, the pharmacological profile of the SR101-labeling in the hippocampus (figure 12) favors an orphan organic anion transporting polypeptide (OATP) as the transporter for selective loading of SR101 into astrocytes. Unfortunately, most of the pharmacology on OATP has been performed with human transporter clones, and thus species differences cannot be excluded for the mouse orthologues. Nevertheless, we can assume that hippocampal astrocytes have a higher expression of that uptake transporter as compared to VLM astrocytes.

### Efflux transporter inhibition does not improve astrocyte labeling in the VLM

It appears very unlikely that multidrug resistance proteins (MRPs) of the ABCC-subfamily or p-glycoprotein are actively lowering the SR101-concentration in the VLM astrocytes, since inhibition of those efflux transporters did not improve SR101-labeling of VLM astrocytes. Neither MK-571 (MRP1, Mrp2) nor probenecid (MRP2-6) did increase the SR101-fluorescence in VLM astrocytes. However, we observed an increase of SR101-staining in EGFP-negative cells, suggesting that MRPs contribute to the de-staining of cells in the VLM.

### A potential role of the orphan SR101-transporter in the hippocampus

It is interesting to note that sulfated neurosteroids, such as dehydroepiandrosterone sulfate (DHEAS) [38] or pregnenolone sulfate [42], are known substrates of organic anion transporting polypeptides. Thus, we can speculate that in the hippocampus, astrocytic organic anion transporting polypeptides might be involved in transport and regulation of neurosteroids and thereby might modulate neuronal network activity [37], [43] or glial plasticity [44], [45]. If this is the case, external application of SR101 could compete with the astrocytic uptake of those neurosteroids and then may cause side effects such as described by Kang et al. [16]. SR101-induced elevation of DHEAS or pregnenolone-sulfate might increase neuronal excitability by blocking GABA_A_ receptors [46], [47], [48] and activation of NMDA-receptors [43], [49].

### Conclusion

In conclusion, the selective labeling of astrocytes in the hippocampus with SR101 was confirmed, but this method is not applicable for the identification of astrocytes in the ventrolateral medulla. Additionally, our data strongly suggest that SR101 is taken up by hippocampal astrocytes via a transporter for organic anions, most likely an organic anion transporting polypeptide (OATP), which, however, is missing or differentially regulated in VLM astrocytes. An unequivocal identification of the candidate gene among the cloned OATPs requires new experiments.

## Supporting Information

Movie S1 **This movie is a two-photon z-stack recording (70 µm, 2 µm, 0.5 min^−1^) of a SR101-staining experiment with 3 different SR101-concentrations.** After recording one z-stack without SR101, 0.1 µM SR101 was bath-applied, 20 minutes later the SR101-concentration was increased to 1 µM for 20 minutes. Finally, 10 µM SR101 was applied for 20 minutes before the recording was stopped. The white spots identify EGFP-labeled astrocytes that were selected for analysis of fluorescence intensities (Figure 5). The blue spot marks the position where fluorescence background within the slice was recorded, while the purple spot was located above the slice to record fluorescence changes of the bath solution (Scale bar 40 µm).
(AVI)
